# Towards Accurate Photogrammetry Using Molded Markers

**DOI:** 10.3390/s24247962

**Published:** 2024-12-13

**Authors:** Iñigo Auzmendi Iriarte, Oier Saez de Egilaz, Pedro Gonzalez de Alaiza Martinez, Imanol Herrera

**Affiliations:** IDEKO Research Center, Basque Research and Technology Alliance (BRTA), 20870 Elgoibar, Spain; osaezdeegilaz@ideko.es (O.S.d.E.); pgonzalezdealaiza@ideko.es (P.G.d.A.M.); iherrera@ideko.es (I.H.)

**Keywords:** accurate photogrammetry, polarimetry, fiducial markers, molded markers

## Abstract

Traditional marker-based photogrammetry systems often require the attachment and removal of a sticker for each measured point, involving labor-intensive manual steps. This paper presents an innovative approach that utilizes raised, cross-shaped markers, referred to as ‘molded markers’, directly embedded into composite pieces. In this study, these markers, commonly employed in other industrial processes, serve as fiducial markers for accurate photogrammetry. A two-stage detection algorithm is developed to accurately identify their centers: initial approximate detection by a Faster R-CNN model, followed by accurate localization using a classical cross center detection algorithm. This study investigates the pertinence of using polarimetric images to guarantee the highest detection rate and accuracy even in adverse lighting conditions. Experimental results demonstrate the viability of using these markers in accurate photogrammetry systems, achieving a median accuracy of 0.170 (interquartile range (IQR) 0.069 to 0.368) mm/m while enhancing automation and system usability.

## 1. Introduction

In accurate photogrammetry, exact localization of keypoints in images directly influences the fidelity of the resulting 3D reconstruction. Traditionally, in marker-based photogrammetry, optical markers, a type of retro-reflective fiducial marker, have been utilized to achieve high levels of measurement precision and accuracy by using their centers as keypoints. However, employing them requires adhering stickers onto the surfaces of objects and scenes to be measured, which can be laborious and time-consuming.

This study explores the use of molded markers, which are created during the fabrication (molding) process of composite pieces. Initially intended to guide specific operations, these markers are integrated into composite parts. This integration not only eliminates the need for the manual application and removal of stickers but also facilitates the use of these markers in photogrammetry systems, particularly within assembly lines. Moreover, as they are designed and placed to be the reference of the operations, it eliminates the need for further referencing of the photogrammetric systems, reducing the uncertainty chain.

The approach also addresses the challenge of applying stickers to pieces with materials that have specific processing properties, which may prevent adhesion. The molded markers, which come in various forms such as crosses and dots, are relief features on the surface of the composite material. This study specifically focuses on cross-shaped molded markers as fiducial markers, aiming to develop a detection system suited for controlled environments, such as those found in industrial settings. The conditions addressed in this work reflect those typically encountered in such environments.

Non-uniform lighting conditions and composite materials introduce complexity into the detection process due to their lack of reflective properties compared to optical targets. This is heightened at distances exceeding 500 mm, a common scenario in photogrammetric applications. To overcome these challenges and effectively leverage the relief texture of the markers, this paper explores the use of polarimetric cameras.

The contributions of this study are as follows:

1. Introduction of a Novel Detection Method: A new, accurate method for detecting the centers of molded markers used in photogrammetry systems is introduced. This method integrates deep learning techniques for initial detection with advanced image processing for accurate center localization.
2. Analysis of Data Augmentation Techniques: Data augmentation techniques that incorporate polarimetric physics directly into camera outputs (intensities) are examined, along with various polarimetric image representations.
3. Enhanced Marker Distinguishability: Improved distinguishability of relief-type markers on composite materials is demonstrated using polarimetric images.
4. 3D Photogrammetric Reconstructions: 3D photogrammetric reconstructions based on these markers are conducted, with results compared to ground truth data obtained from a laser tracker to assess the accuracy of the system.

## 2. Related Work

This section presents a comprehensive review of the relevant literature on photogrammetry systems, with a particular focus on the various types of markers utilized in these systems. Following the discussion on markers and their detection, it also examines advancements in object detection methods, which are critical for their localization. Additionally, it explores the role of polarimetry as an enabling technology for detecting accurately molded markers, its applications across different fields, and the integration of polarimetric images with neural networks.

### 2.1. Photogrammetry Systems

Photogrammetry entails accurately measuring the 3D coordinates of keypoints using various perspective images [1] reconstructed by triangulation [2]. This can be achieved using multiple fixed cameras or a portable system with a single camera that is moved to capture different viewpoints. In the domain of accurate industrial metrology, especially in large volumes, it has emerged as one of the preferred choices due to its versatility and relatively lower investment requirements [3].

#### Markers

Photogrammetry systems involve detecting keypoints on the objects to be measured. These keypoints can be identified using a variety of methods, which are broadly categorized into marker-less techniques and fiducial marker-based approaches.

Marker-less techniques leverage natural features in the images. Traditional approaches often rely on natural descriptors, such as corners or texture patterns, for keypoint detection [4,5]. However, their effectiveness is limited in industrial environments, where objects may lack sufficient texture or distinctive features. Structured light-based approaches address this challenge by projecting patterns onto the object to facilitate keypoint detection [6,7]. While these techniques can be highly effective, their accuracy depends heavily on the quality of the projection system, which introduces high costs and system complexity, limiting their applicability in certain scenarios. Alternatively, other marker-less techniques use specific edges or geometric features as references to define virtual keypoints. For example, some methods analyze the relative positions of edges to estimate motion and vibrations, as seen in rotating cylindrical structures [8] or wind turbine blades [9]. These methods are typically limited to easily detectable edges, making them more suited for motion analysis than dimensional reconstruction. Furthermore, Artificial Intelligence (AI)-based approaches for keypoint estimation have emerged. In applications like railway maintenance, AI models have been used to estimate predefined keypoints for displacement measurements [10]. However, the accuracy of these AI models remains insufficient.

Thus, when high dimensional accuracy is required, fiducial marker-based approaches remain the preferred method [3]. Among these, optical marker stickers are the most commonly used due to their retro-reflective properties, which allow for clear differentiation from surrounding objects [11,12]. However, this approach requires the application and removal of stickers, which is time-consuming and impractical for automatic processes. To avoid the need for stickers, mechanical markers have been explored. One study introduced a novel design using CNC-machined components with two holes of varying diameters as fiducial markers [13]. However, creating such markers is not always feasible, as it requires modifications to the measured object and can be time-consuming.

### 2.2. Object Detection

Object detection, a core task in computer vision, involves identifying and localizing objects within an image. Detecting fiducial markers, especially complex ones like the molded markers in this work, often requires advanced methods. Neural networks, particularly Convolutional Neural Networks (CNNs) and transformers, have significantly advanced object detection. Methods are typically categorized as one-stage or two-stage approaches: one-stage models directly predict bounding boxes and classes in a single step, offering speed, while two-stage models first identify regions of interest for classification, prioritizing accuracy.

#### 2.2.1. CNNs

CNN-based one-stage detectors, such as Single Shot Detector (SSD) [14] and You Only Look Once (YOLO) [15], along with their various iterations, have revolutionized object detection by offering faster inference speeds, making them suitable for real-time applications while maintaining acceptable accuracy. On the other hand, two-stage methods generally achieve higher accuracy and have been extensively studied. For instance, Faster R-CNN [16] is well known for its popularity, superior accuracy, and reduced training and inference times compared to earlier models like R-CNN [17] and Fast R-CNN [18].

#### 2.2.2. Transformers

More recently, transformer-based models leveraging attention mechanisms have redefined the field of computer vision, often outperforming traditional CNNs. In object detection, one-stage models like DETR [19] and its successor Deformable DETR [20] have achieved state-of-the-art results. However, these architectures require substantial amounts of training data to outperform CNN-based methods, limiting their suitability for industrial applications where data availability is often restricted.

A comprehensive overview of the evolution of object detection models introduced in recent years can be found in [21].

### 2.3. Polarimetry

Light, as an electromagnetic wave, exhibits three primary physical elements: amplitude, representing brightness; wavelength, indicating colors; and polarization, denoting the direction of wave oscillation [22]. Unpolarized light, like sunlight or fluorescent lamp emissions, oscillates in multiple directions, whereas polarized light oscillates exclusively in one direction.

**Figure 1:**
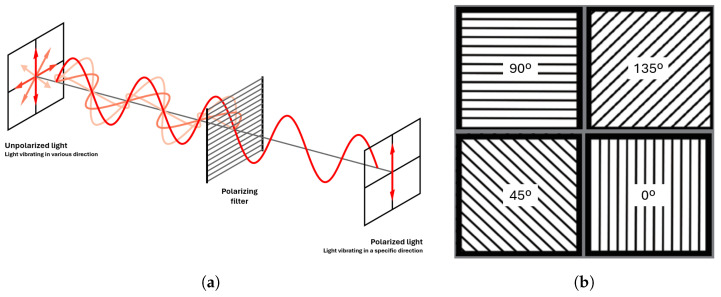
Usage of polarization filters in cameras. (**a**) Effect of a polarization filter on unpolarized light. (**b**) Distribution of superpixel polarization filters in cameras (each camera pixel contains four different polarization intensity values). The angle indicates the polarization angle associated with each filter.

Polarization filters in digital cameras selectively pass rays of specific polarization angles, polarizing non-polarized light upon passage (Figure 1a). When non-polarized light reflects off a material, it generates partially polarized rays depending on the surface normal and material refractive index. Polarimetric cameras typically employ polarizers at angles of $0^{\circ }$, $45^{\circ }$, $90^{\circ }$, and $135^{\circ }$, giving at each pixel the corresponding intensities (Figure 1b). From them, as shown by Equation (1), Stokes parameters, $S_{0}$, $S_{1}$, and $S_{2}$ can be derived. Specifically, $S_{0}\geq 0$ denotes total light intensity, akin to a grayscale image. $S_{1}$ captures the excess polarized light intensity between horizontal and vertical filters, while $S_{2}$ gives the surplus between $45^{\circ }$ and $135^{\circ }$ ones. These parameters define the intensity profile (cf. Equation (2)), and the sum of intensities at two polarizers separated by $90^{\circ }$ equals $S_{0}$ (cf. Equation (3)). Moreover, the Angle of Polarization (ϕ) and the Degree of Polarization (ρ) are derived from the Stokes parameters. They indicate the orientation of polarized light (cf. Equation (4)) and the intensity of polarized light relative to the total intensity (cf. Equation (5)) [23].

(1) $$S=\left[\begin{array}{c} S_{0}\\ S_{1}\\ S_{2} \end{array}\right]=\left[\begin{array}{c} \frac{1}{2}\left(I_{0}+I_{90}+I_{45}+I_{135}\right)\\ I_{0}-I_{90}\\ I_{45}-I_{135} \end{array}\right]$$

(2) $$I_{\alpha }=\frac{1}{2}\left(S_{0}+S_{1}\cos2\alpha +S_{2}\sin2\alpha \right),$$

(3) $$I_{0}+I_{90}=I_{45}+I_{135}=S_{0},$$

(4) $$\phi =\frac{1}{2}\arctan\left(\frac{S_{2}}{S_{1}}\right)\in \left[-\frac{\pi }{2},\frac{\pi }{2}\right],$$

(5) $$\rho =\frac{\sqrt{S_{1}^{2}+S_{2}^{2}}}{S_{0}}\in \left[0,1\right].$$

#### 2.3.1. Use of Polarimetric Images

Polarimetric imaging has been extensively used to differentiate between specular and diffuse reflections [24,25] or to eliminate these reflections entirely [26]. This distinction arises due to surface inconsistencies: light reflected from rough surfaces generates unpolarized light (diffuse reflections), whereas smooth surfaces produce more polarized light (specular reflections). This polarization property has been leveraged in various applications, such as enhancing contrast in underwater imaging [27] and segmenting transparent objects [28]. Moreover, by analyzing polarization from multiple views, ambiguities in surface normal estimation can be resolved, enabling the generation of depth maps [29] and aiding in pose estimation [30].

Additionally, because polarization inherently contains information about surface normals, it has been widely applied in surface defect detection, such as in steel strip inspection [31] and composite material evaluation [32]. Molded markers, as irregularities on surfaces, can be interpreted as defects, and polarization techniques can be particularly effective in detecting and localizing them.

#### 2.3.2. Neural Networks Fed by Polarimetric Images

When integrating polarization information into neural networks, it is imperative to pre-process the data appropriately to align with the network architecture. Several representations of polarization data have been proposed (Table 1), each presenting distinct advantages tailored to specific applications.

The authors of [23] found that representations using three intensities, Stokes, and Pauli-style, were optimal for road scene analysis. Conversely, Ref. [28] demonstrated that fusing the HSL-like representation with attention layers yielded the best results in transparent object detection. Similarly, Ref. [34] achieved superior performance in urban scene understanding tasks by leveraging the HSL-like representation. Additionally, Ref. [33] utilized polarimetric images with a quarter-wave plate to have a four-component Stokes vector to reconstruct scenes in 3D polarimetric images, surpassing other representations in detection tasks, even in challenging conditions like low light levels and partial occlusion of targets. Overall, while these studies highlight the benefits of integrating polarization information into neural networks through appropriate data representations, the choice of representation appears to be dependent on the specific domain, with no universally preferred option.

In order to address the limited amount of data, numerous studies have investigated data augmentation techniques tailored specifically for polarimetric images, focusing on physically realistic transformations to improve neural network performance. The literature claims that applying a comprehensive transformation of polarimetric values, prior to flipping and rotation transformations, can notably enhance dataset generalization and performance [28,34]. For instance, when rotating images by an angle θ, adjusting the corresponding ϕ value by subtracting θ has shown benefits, as well as multiplying ϕ values by −1 for flipped images. These methodologies capitalized on polarization properties in those works, yielding superior outcomes compared to traditional transformations.

## 3. Materials and Methods

### 3.1. Dataset

The images that comprised the dataset used in the methodology were captured using a FLIR Blackfly S BFS-PGE-123S6P-C camera [35], featuring a resolution of 2048×1500 superpixels with angles at $0^{\circ },45^{\circ },90^{\circ }$, and $135^{\circ }$. It was complemented by a Computar V2520-MPZ lens with a focal length of 25 mm. A DCM ALB1716A-W00i/AN white, narrow-angle ring light was used during image acquisition.

**Figure 2:**
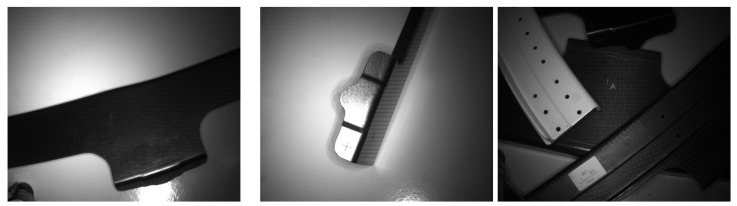
Examples of images from the dataset utilized in this study.

The dataset comprises 196 annotated images of Carbon Fibre-Reinforced Polymer (CFRP) composite pieces, each containing at least one molded marker, taken at a working distance of 500–600 mm. The fixed size of the molded cross markers is 10×10 mm, with a height of 1.5 mm and a cross thickness of 0.5 mm. Various examples of images from the dataset are depicted in Figure 2. Due to the material properties and the positioning of the lighting, the central markers are better illuminated than those farther from the center.

Overall, the dataset was divided into three subsets: the training set (65%) to fine-tune the Faster R-CNN detection model; the validation set (15%) to select hyperparameters and the final model instance; and the test set (20%) to test and validate the generalization of the trained model. The dataset contains 471 instances of molded markers of a mean size of 35×35 pixels.

Despite employing an exposure time of 60 ms (lower exposure times resulted in insufficient illumination, while higher exposure times increased noise), high noise was observed in the polarimetric images, as previously noticed in [36,37], which could adversely affect mostly the accurate center detection. To mitigate the issue, each image in the dataset was denoised by averaging consecutive images. This method effectively reduced the noise generated by the polarimetric technology of the lens.

### 3.2. Detection System

**Figure 3:**
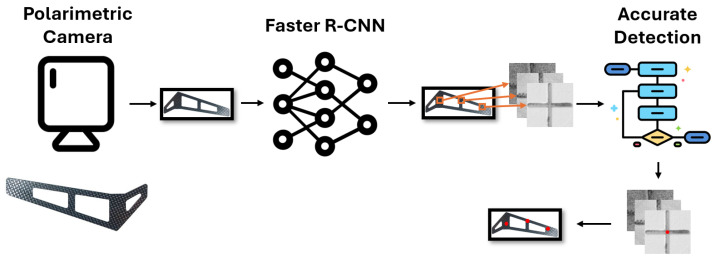
Proposed molded marker center detection pipeline. Firstly, an image is captured using a polarimetric camera. Afterwards, molded markers are detected within the image using the Faster R-CNN model, followed by the determination of their centers using an accurate cross center detection algorithm.

A two-step approach was employed to accurately detect the centers of molded markers, as illustrated in Figure 3. First, a neural network was trained/fed by a polarimetric representation to detect candidates as seeds for the second step. Faster R-CNN was selected because of its wide adoption, accessibility, and ease of implementation at the time.

**Figure 4:**
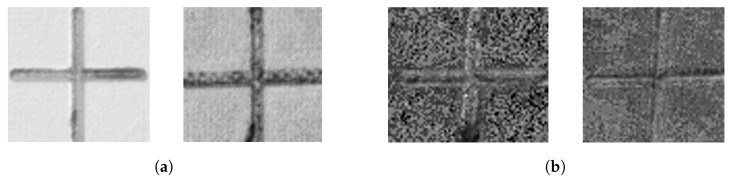
Examples of molded marker crops retained and discarded by the accurate center detection algorithm. (**a**) Retained crops. (**b**) Discarded crops.

Second, accurate detection of the marker center on a polarimetric image was carried out using classical computer vision techniques, specifically customized for cross markers. Initially, the image undergoes binarization using the Sauvola method [38] after pre-processing. Next, segments are detected through the Hough transform [39] and their corresponding directions are organized in a histogram. Then, two groups of segments in the image are formed, according to the two most prominent and most separated directions in that histogram. The axes of the cross are finally obtained through least-square fitting, weighting by segment lengths. Finally, the binarized image is compared with the synthetic ideal reconstructed cross from the calculated axes to decide whether the marker is retained or discarded. If the comparison succeeds, the obtained center is the intersection of the axes. Examples of discarded and retained molded marker crop images are illustrated in Figure 4.

In this system, markers that are excessively blurred or occluded are discarded. The approximate detection network was specifically trained to identify only complete and high-quality markers, with markers that were severely occluded or blurry excluded from annotation during training. Moreover, in the final step of the accurate center detection process, markers that are either occluded or blurred are filtered out based on a comparison between the synthetic ideal binarized image and the real binary image. Occluded markers are missing parts of the marker, while blurry markers result in excessively wide lines in the binary image. Although the material and size of the markers minimize major deformations, the ellipse-fitting detection method employed in the accurate detection phase is robust enough to determine the center of markers even with minor deformations.

### 3.3. Data Augmentation Polarimetric Techniques

Unlike previous approaches, which primarily focused on manipulating the ϕ field, in this work these extra transformations will be tested, locally performed directly on original intensity fields $I_{0}$, $I_{45}$, $I_{90}$ and $I_{135}$.

#### 3.3.1. Image Flip

Flipping the image, either horizontally or vertically, will physically interchange the values of the channels $I_{45}$ and $I_{135}$ seen by the camera. Therefore, before the conventional flip transformation of the image, it is required to permute locally those intensity values per pixel.

#### 3.3.2. Image Rotation

Solely rotations of the images around their optical axis are considered here. If the sensor is physically rotated around the target through an angle −θ, then the local polarimetric intensity profiles need to be translated by the opposite angle, θ, as follows (four- and three-channel versions are shown):

(6) $${\left[\begin{array}{c} I_{0}\\ I_{45}\\ I_{90}\\ I_{135} \end{array}\right]}^{\mathrm{rot},\theta }=\left[\begin{array}{cccc} \frac{1}{4}+\frac{1}{2}\cos2\theta & \frac{1}{4}+\frac{1}{2}\sin2\theta & \frac{1}{4}-\frac{1}{2}\cos2\theta & \frac{1}{4}-\frac{1}{2}\sin2\theta \\ \frac{1}{4}-\frac{1}{2}\sin2\theta & \frac{1}{4}+\frac{1}{2}\cos2\theta & \frac{1}{4}+\frac{1}{2}\sin2\theta & \frac{1}{4}-\frac{1}{2}\cos2\theta \\ \frac{1}{4}-\frac{1}{2}\cos2\theta & \frac{1}{4}-\frac{1}{2}\sin2\theta & \frac{1}{4}+\frac{1}{2}\cos2\theta & \frac{1}{4}+\frac{1}{2}\sin2\theta \\ \frac{1}{4}+\frac{1}{2}\sin2\theta & \frac{1}{4}-\frac{1}{2}\cos2\theta & \frac{1}{4}-\frac{1}{2}\sin2\theta & \frac{1}{4}+\frac{1}{2}\cos2\theta \end{array}\right]\left[\begin{array}{c} I_{0}\\ I_{45}\\ I_{90}\\ I_{135} \end{array}\right]$$

(7) $${\left[\begin{array}{c} I_{0}\\ I_{45}\\ I_{90} \end{array}\right]}^{\mathrm{rot},\theta }=\left[\begin{array}{ccc} \cos\theta (\cos\theta -\sin\theta ) & \sin2\theta & \sin\theta (\sin\theta -\cos\theta )\\ \sin\theta (\sin\theta -\cos\theta ) & \cos2\theta & \sin\theta (\sin\theta +\cos\theta )\\ \sin\theta (\sin\theta +\cos\theta ) & -\sin2\theta & \cos\theta (\cos\theta +\sin\theta ) \end{array}\right]\left[\begin{array}{c} I_{0}\\ I_{45}\\ I_{90} \end{array}\right].$$

Equation (6) is derived as follows. The rotated intensities are expressed as a function of the Stokes parameters by giving values α=θ, α=π/4+θ, α=π/2+θ, and α=3π/4+θ in Equation (2). The Stokes parameters are, in turn, related to the unrotated intensities thanks to Equation (1), finally obtaining Equation (6) after some manipulations with matrices. Equation (7) is derived from Equation (6), after applying Equation (3).

Note that the matrix in Equation (6) is singular because one of the intensities is linearly dependent on the rest according to Equation (3). Immediately after this local transformation of pixel values, the conventional rotation transformation would be applied to the image. However, the angle for this rotation actually does not need to be equal to −θ, in the case that eventual relative rotations between the lighting and the camera are considered.

**Figure 5:**
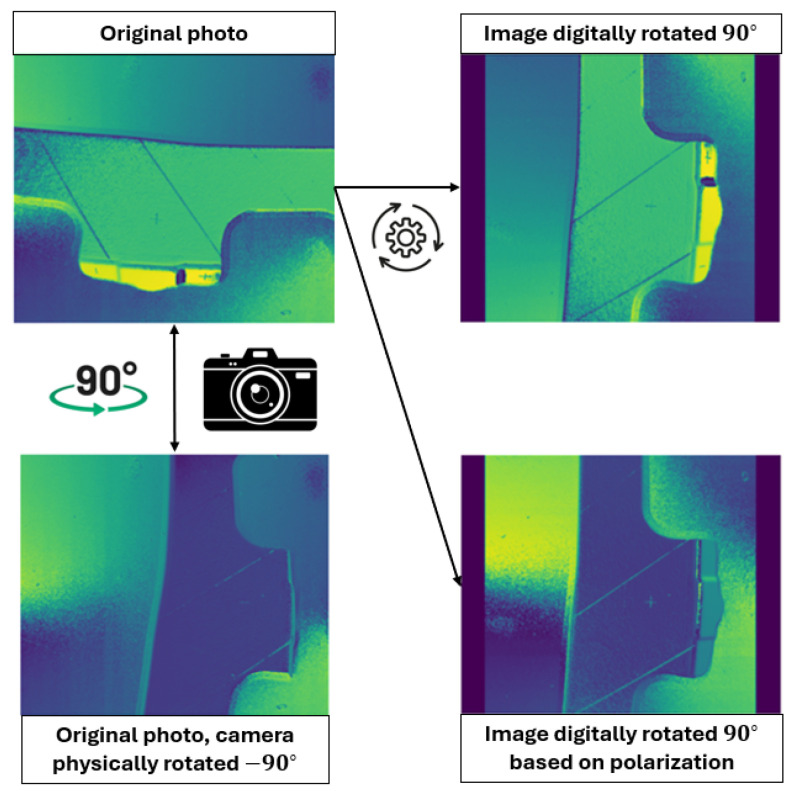
Original image ϕ, results from applying conventional and polarization-based rotation to the original image and the ϕ obtained by physically rotating the camera.

Figure 5 illustrates that utilizing polarization-based transformations yields an image closer to the physically rotated one compared to conventional rotation transformations for $\theta =90^{\circ }$.

## 4. Experiments

### 4.1. Implementation and Execution Environment

For the approximate detection, training was conducted using the PyTorch library in Python using a cloud solution with an NVIDIA T4 GPU. The molded marker center detection, on the other hand, was developed using Python in conjunction with the OpenCV library purely on the CPU.

The detection experiments were executed in a system equipped with an Intel Core Ultra 9 185H CPU (22 threads) with 32 GB RAM. The approximate marker detection stage was executed on the CPU, as the primary focus was to achieve accuracy before optimizing for system efficiency.

### 4.2. Approximate Detection

The Faster R-CNN model with a FPN-ResNet-50 backbone was selected due to its superior small object detection capabilities [40], which aligns with the dimensions of the molded markers in our images. Moreover, its effectiveness in mitigating vanishing gradients in deep networks is recognized, offering performance comparable to larger backbones with improved inference time [41]. Training utilized a pre-trained Faster R-CNN model from the COCO dataset [42] as the basis, with image sizes reduced to 1224×1024 to accommodate GPU memory and training time constraints. The training process employed a batch size of eight with the Adam optimizer [43] and a learning rate of $10^{-5}$, lasting for 100 epochs, as additional epochs did not significantly improve training outcomes.

Different training experiments were conducted to provide insights into how different data augmentation strategies, including conventional and polarization-preserving rotations and flips, impact the training behavior of the model. Initially, training was conducted without any data augmentation to establish a baseline result. Subsequently, conventional rotation (random angle) and flip (50% horizontal and vertical flip) transformations were applied. Following this, the same transformations were applied while preserving the polarization physics, as explained in Section 3.3. After that, both conventional and polarization-preserving transformations were combined. This involved combining both types of transformations, where the angle of polarization rotation could differ from the angle used to rotate the image, and polarization could be flipped horizontally and vertically while keeping the image unchanged, and vice versa.

In addition to the data augmentation techniques, considering the previous literature’s indication that different representations of polarimetric images are optimal for different applications, this study compared various representations aligned with the requirement of the pre-trained model, typically trained on RGB images (three channels). The explored representations included three intensities ($I_{0}$, $I_{45}$, and $I_{90}$), Stokes ($S_{0}$, $S_{1}$, $S_{2}$), and the HSL-like representation (ϕ, ρ, and $S_{0}$). Subsequently, training was conducted using only the $S_{0}$ channel to emulate a conventional grayscale image, enabling a comparison of detection outcomes without leveraging polarimetry. Additionally, a final training iteration was performed using only ρ, as it was presumed to be the representation where the cross markers were most distinguishable from the background.

To assess the performance of the trained detection model, the mean Average Precision at 50% Intersection over Union (IoU) metric ($mAP_{50}$) was employed [44]. This metric is widely used in object detection tasks to evaluate the accuracy of predicted bounding boxes by comparing them to the ground truth based on the degree of overlap (IoU). It combines precision and recall to assess detection performance. In this study, an IoU threshold of 50% was selected, meaning that a predicted bounding box is considered correct if its overlap with the ground truth is at least 50%. This choice aligns with the objective of the approximate detection phase, which focuses on identifying markers and obtaining approximate localization information. Given the small size of some markers, with higher IoU values, minor differences of just a few pixels could cause detections to be classified as incorrect, despite being sufficient to seed the next accurate step.

### 4.3. Accurate Center Detection

The detection rate was calculated for each polarimetric image type to evaluate the ease of marker distinguishability. This experiment was conducted using denoised mean images, as well as with the original noise ones, to assess how noise affected the accurate center detection rate.

### 4.4. Three-Dimensional Accurate Photogrammetric Reconstruction of Markers

The final experiment aimed to validate the system’s performance in a real-world setting and assess its detection accuracy. For this purpose, an aeronautic component (approximately 3600 × 1300 mm) was used, and the distances between the molded markers on it were measured within an industrial environment.

To accurately locate each molded marker in 3D space, a polarimetric camera was securely mounted on a platform attached to the gripper of a KUKA IIWA robotic arm. The robotic arm, positioned on a linear track, was able to move the camera close to each molded marker on the CFRP piece. The arm captured five photographs of each marker from different perspectives, with each image taken at a minimum distance of 500 mm from the marker. Capturing images from multiple angles enabled the system to triangulate the 2D centers of each marker, allowing for accurate 3D photogrammetric reconstruction of their center positions.

**Figure 6:**
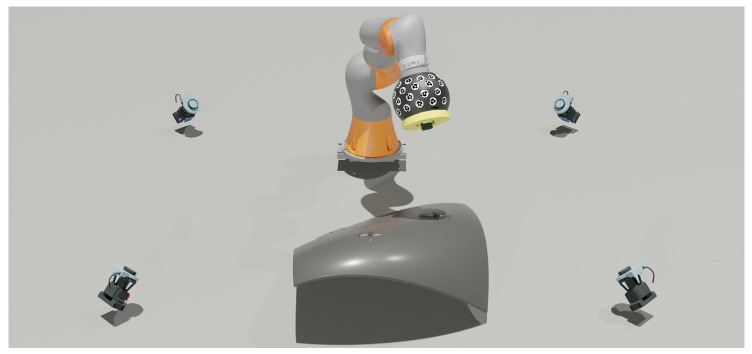
Illustration of the actual photogrammetric scenario used. A four-camera multi-camera system tracks the object on the robotic arm containing optical coded markers, which holds the polarimetric camera.

To determine the 3D positions of the molded markers within the world reference frame, the pose of the robotic arm’s gripper was tracked by a commercially available accurate four-camera photogrammetric system [45] (see Figure 6), which has a stated accuracy of 0.045 mm. Optical coded markers were attached to the gripper to enable precise tracking by this multi-camera system. An eye–hand calibration was then conducted to establish the spatial relationship between the camera and the robotic arm’s gripper.

The process for obtaining the 3D position of each molded marker involved three main steps: first, capturing five images of each marker to calculate its 3D position within the camera’s reference frame; next, applying eye–hand transformation to convert these positions to the gripper’s reference frame; and finally, using the four-camera photogrammetric system to track the gripper’s global position, which ultimately allowed for mapping the final 3D positions of the molded markers to the world reference frame.

**Figure 7:**
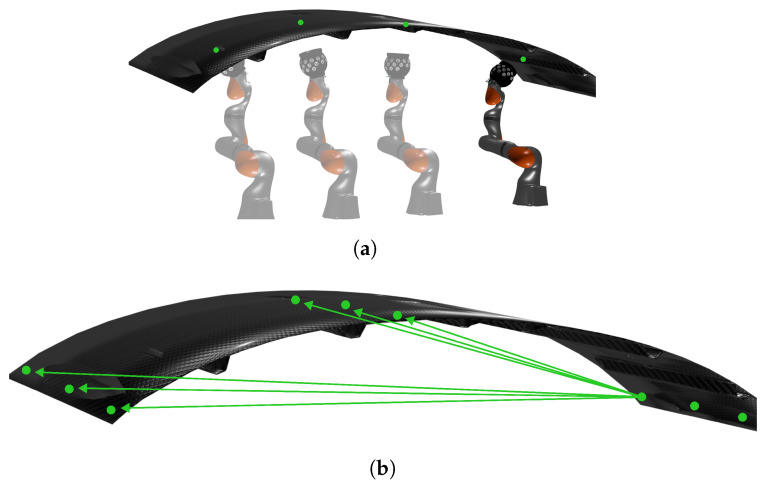
Illustration of the measurement process on the component. (**a**) Robotic arm measuring molded markers on the component. (**b**) Examples of measured distances.

Seventeen markers spread across the CFRP piece were measured (see Figure 7), and the distances between each pair of markers were compared with those obtained from a Leica AT960 laser tracker, which provided the 3D ground truth measurements for the experiment. Only distances exceeding one meter were considered to ensure the results are representative of practical large-scale usage scenarios. The median, interquartile range (IQR), and uncertainty with k=2 were calculated to assess the system’s accuracy based on the 22 measured distances. The measurements were conducted four times to ensure the absence of any significant precision issues.

## 5. Results

### 5.1. Approximate Detection

The diverse outcomes obtained from employing various input representations and applying different data augmentation techniques are summarized in Table 2. Overall, the model achieves a $mAP_{50}$ exceeding 0.9 in most cases, demonstrating that it is sufficient for obtaining nearly perfect results in approximate detection. This level of performance suggests that exploring newer models or alternative techniques to further enhance accuracy is unnecessary. The inference time for the approximate detection stage was approximately 1 s per image when executed on the CPU.

Interestingly, the application of any data augmentation technique appeared to enhance training, likely due to the relatively small size of the dataset used in this study. However, there were no significant differences observed among the results obtained from employing either conventional transformations, polarization-preserving ones, or combining both. Given the small size of the test set, differences around 4% may not be deemed significant, as they could be influenced by non-deterministic training outcomes. This suggests that achieving excellent outcomes with conventional transformations may render additional techniques redundant in this particular scenario.

Finally, the results indicated that input representation is not a critical factor in this dataset, as all approaches yielded similar outcomes. Particularly noteworthy is the effectiveness of using only $S_{0}$ (closest to a standard grayscale camera image) and applying conventional transformations, which implies that polarization physics may not substantially contribute to the approximate detection task.

### 5.2. Accurate Center Detection

The accurate center detection rates obtained using each polarimetric image are detailed in Table 3. Firstly, denoising by averaging ten consecutive images improves the center detection rate in any polarimetric image, showing an improvement of around 10% in the best case. Secondly, the polarization-based ρ image exhibited a 79% improvement in the detection rate compared to the common grayscale image, represented by $S_{0}$, significantly outperforming all other polarization-based images. The execution time for the accurate detection stage was approximately 40 ms per marker.

**Figure 8:**
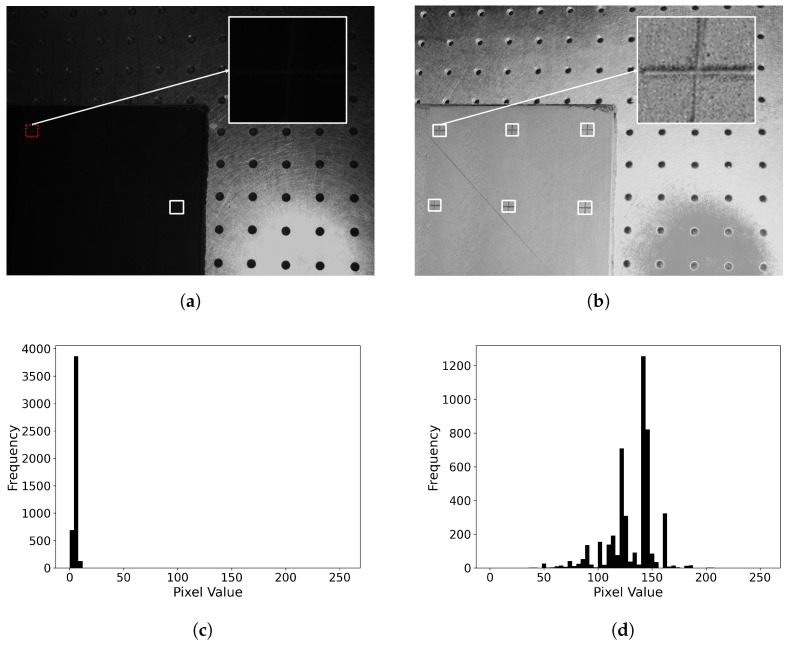
Molded markers with accurately detected centers highlighted in white within the corresponding images (every molded marker was successfully detected by the Faster R-CNN model). (**a**) $S_{0}$ image and crop. (**b**) ρ image and crop. Additionally, the top-left crop of a molded marker under imperfect illumination is shown, along with its corresponding histogram. (**c**) $S_{0}$ marker crop histogram. (**d**) ρ marker crop histogram.

Figure 8a,b compare the accurate detection of crosses’ centers using $S_{0}$ and ρ.

All the markers were successfully detected by the approximate stage. However, while $S_{0}$ performs satisfactorily under direct or nearby light conditions, it struggles with markers situated farther from the light source, resulting in inferior illumination. This limitation was effectively overcome by employing the ρ image, which captures more nuanced information owing to its broader value range and clearer differentiation between the marker and background. Additionally, the histograms of $S_{0}$ and ρ values for the top-left crop in Figure 8c,d further underscore the advantages of utilizing the ρ image under conditions of less-than-ideal illumination. Their analyses reveals a stark contrast in pixel value distribution. The $S_{0}$ histogram shows a limited range of values, while the ρ one demonstrates a wider spread, suggesting richer information for marker center detection.

### 5.3. Three-Dimensional Accurate Photogrammetric Reconstruction of Markers

**Figure 9:**
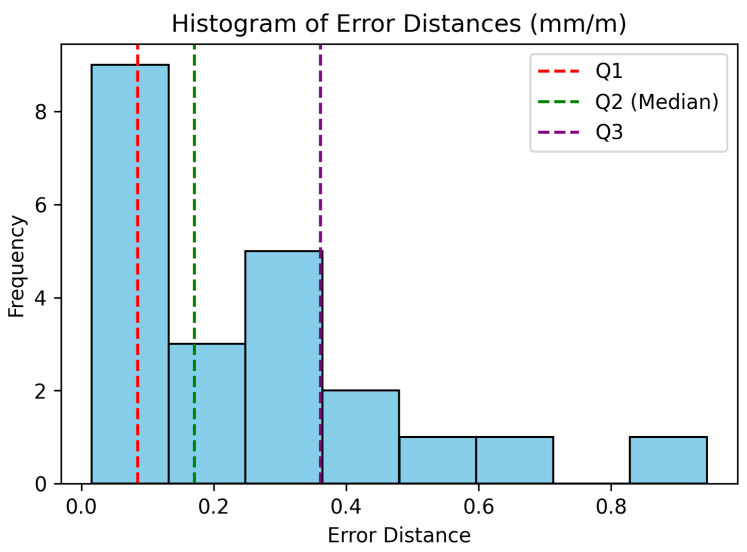
Histogram of absolute distance errors obtained between the photogrammetric system with molded markers and the laser tracker.

The experimental results revealed a median accuracy of 0.17 mm/m (IQR: 0.069 to 0.368 mm/m) or 0.463 mm/m of uncertainty at k=2. The histogram of errors is depicted in Figure 9. This system demonstrates an accuracy one order of magnitude worse than other optical marker-based systems [46]. Despite this, at this initial research stage, the presented concept exhibits enhancements in usability and automation, and it would already be suitable for various industrial applications such as alignment, material handling, assembly of large components, or even coating.

## 6. Discussion

This study introduces the innovative use of molded markers for photogrammetry measurements, and it also explores the use of polarization-based images throughout the center detection process.

Results indicate that, while polarization representations and data augmentation did not significantly enhance detection performance in the initial phase compared to grayscale images with standard transformations, they played a crucial role in subsequent accurate center localization. Particularly, the polarization-based ρ image significantly boosted detection rates by 79%, offering higher quality data, especially in suboptimal lighting conditions. Polarimetric image noise reduction also resulted in an improvement in the center detection rate.

The experiment conducted in a real photogrammetric scenario confirmed the viability of molded markers for accurate photogrammetric measurement systems. The accuracy obtained is suitable for various industrial applications, including alignment, drilling, machining guidance, and the tracking of composite pieces.

This methodology has certain limitations, as the material molding process must be compatible with embedding the molded markers. Additionally, this approach is unsuitable for pieces where the final finish must remain unaltered, as the molded markers would modify the original surface. In such cases, a post-processing step would be required to remove the molded markers, which may be time consuming. Furthermore, while molded markers can tolerate some deformation, they are not suitable for areas with extreme deformations or highly curved surfaces, as this could hinder their detection.

Future research will focus on refining the accuracy of the cross center detection algorithm to enhance 3D measurement accuracy, potentially through the integration of classical filters or machine learning techniques. Additionally, future research should explore the potential of leveraging polarization physics, such as utilizing polarization angles to detect the peaks of relief on the markers. This approach could enable a more refined delineation of marker edges, ultimately improving detection accuracy. Furthermore, the detection system will be expanded to incorporate the capability to identify and differentiate between various shapes, thereby increasing its flexibility. To achieve this, additional physical samples will be produced, and the training dataset will be appropriately augmented. Expanding the system’s capacity to operate over larger volumes is also a key goal to broaden its applicability.

The industrialization of the system will necessitate optimizing both stages of the detection process, including the adoption of newer and more efficient models for the approximate detection stage. Furthermore, leveraging the detected centers as ground truth annotations for keypoints and exploring direct center detection through alternative neural network architectures, such as keypoint detectors, could further streamline the process and enhance efficiency. The total detection execution time after optimizing should be around 100 ms, which aligns with commonly used optical detection algorithms [47]. This target speed is crucial for adopting molded markers in industrial settings without affecting performance.

In applications involving uncontrolled environments with extreme lighting conditions or high noise levels, further investigation into advanced filtering techniques will be essential to improve detection robustness.

## Figures and Tables

**Table 1:** Different detection model input representations of polarimetric images.

Data Format	Channel 1	Channel 2	Channel 3	Channel 4	Used in Article
Int. (3 ch)	$I_{0}$	$I_{45}$	$I_{90}$	-	[23]
Int. (4 ch)	$I_{0}$	$I_{45}$	$I_{90}$	$I_{135}$	[28]
Stokes	$S_{0}$	$S_{1}$	$S_{2}$	-	[23,33]
Pauli	$S_{0}$	$S_{1}$	$I_{45}$	-	[23]
HSL-like	ϕ	ρ	$S_{0}$	-	[23,28,34]
Poincaré	$S_{0}$	ρcos(2ϕ)	ρsin(2ϕ)	-	[23]

**Table 2:** Test set $mAP_{50}$ detection results after training with different input representations and different data augmentation techniques. Bold values indicate the best-performing data augmentation method for each input representation.

	$I_{0}$, $I_{45}$, $I_{90}$	$S_{0}$, $S_{1}$, $S_{2}$	ϕ, ρ, $S_{0}$	$S_{0}$	ρ
No data augmentation	0.843	0.79	0.875	0.862	0.828
Conventional transformations	**0.96**	0.925	0.946	0.926	0.915
Polarization based transformations	0.922	0.925	**0.954**	**0.938**	**0.924**
Conv. + Polariz. based transforms.	0.94	**0.936**	0.939	0.935	0.913

**Table 3:** Accurate center detection rate of different polarimetric images using the average of 10 images and using just 1 original image. Bold values highlight the best-performing polarimetric image and the highest detection rate achieved.

Image Used	Acc. Center Det. Rate	
	Avg. of 10	Single Img.
$I_{0}$	0.1	0.06
$I_{135}$	0.59	0.56
$I_{45}$	0.36	0.33
$I_{90}$	0.59	0.57
$S_{0}$	0.48	0.47
$S_{1}$	0.15	0.13
$S_{2}$	0.01	0.006
ϕ	0.09	0.03
ρ	**0.86**	0.79

## Data Availability

The dataset presented in this article is not available due to privacy and commercial restrictions.
